# Personality Factors and Suicide Risk in a Representative Sample of the German General Population

**DOI:** 10.1371/journal.pone.0076646

**Published:** 2013-10-04

**Authors:** Victor Blüml, Nestor D. Kapusta, Stephan Doering, Elmar Brähler, Birgit Wagner, Anette Kersting

**Affiliations:** 1 Department of Psychoanalysis and Psychotherapy, Medical University of Vienna, Vienna, Austria; 2 Department of Medical Psychology and Medical Sociology, University of Leipzig, Leipzig, Germany; 3 Integrated Research and Treatment Center (IFB) Adiposity Diseases, Leipzig University Medical Center, Leipzig, Germany; 4 Department of Psychosomatic Medicine and Psychotherapy, University of Leipzig, Leipzig, Germany; McGill University, Canada

## Abstract

**Objective:**

Previous research has shown an association between certain personality characteristics and suicidality. Methodological differences including small sample sizes and missing adjustment for possible confounding factors could explain the varying results. The aim of this study was to assess the impact of the Big Five personality dimensions on suicidality in a representative population based sample of adults.

**Method:**

Interviews were conducted in a representative German population-based sample (n=2555) in 2011. Personality characteristics were assessed using the Big Five Inventory-10 (BFI-10) and suicide risk was assessed with the Suicidal Behaviors Questionnaire-Revised (SBQ-R). Multivariate logistic regression models were calculated adjusting for depression, anxiety, and various sociodemographic variables.

**Results:**

Neuroticism and openness were significantly associated with suicide risk, while extraversion and conscientiousness were found to be protective. Significant sex differences were observed. For males, extraversion and conscientiousness were protective factors. Neuroticism and openness were found to be associated with suicide risk only in females. These associations remained significant after adjusting for covariates.

**Conclusion:**

The results highlight the role of personality dimensions as risk factors for suicide-related behaviors. Different personality dimensions are significantly associated with suicide-related behaviors even when adjusting for other known risk factors of suicidality.

## Introduction

Suicide is a major public health issue accounting for over one million deaths per year making it the tenth leading cause of death worldwide [1]. Known risk factors include age, sex, presence of psychiatric disorders, and other sociodemographic characteristics such as religious denomination and unemployment [1,2].

An increasing body of evidence suggests an association between certain personality factors and suicidality [3]. Past research applied a variety of different concepts of personality complicating definite conclusions about the influence of personality traits on suicidality [3]. The five-factor model of personality is one of the most comprehensive and best established models to assess personality [4,5]. It aims to empirically describe personality along five major dimensions, namely neuroticism, extraversion, openness, agreeableness, and conscientiousness [4,5]. These five personality dimensions show clear heritable characteristics [6,7] and have been shown to be associated with different psychiatric disorders such as anxiety, depressive, substance use, and personality disorders [8,9,10,11].

In a systematic review of the association between personality traits and suicide risk, Brezo et al. (2006) found neuroticism and extraversion to be the most consistently replicated characteristics to be associated with suicide-related behaviors. Specifically, higher neuroticism was associated with suicidal ideation, suicide attempts, and completed suicide, while extraversion was found to be a resilience factor for suicidality [3]. In a clinical sample of depressed adults 50 years of age or older, higher scores of neuroticism and openness were associated with suicidal ideation [12]. Similar results in another sample of depressed adults 50 years of age or older were reported for the association between personality facets (sub-scales of the Big Five dimensions) and suicide attempts [13]. High neuroticism and low extraversion together with low agreeableness and low conscientiousness were reported to be associated with increased suicidal ideation in a college student sample [14], whereas only neuroticism was found to be a predictor for suicide ideation in another sample of university students [15]. Additionally, the influence of personality traits on suicidality was found to be influenced by gender [16]. In a sample of young adults, neuroticism was associated with suicidal ideation in females, while conscientiousness was negatively associated with suicidal ideation in males [17]. In one of the few studies investigating personality characteristics and completed suicide, a personality style characterized by a combination of high neuroticism and low extraversion was found to increase suicide risk in an adult sample in rural China [18].

However, a major limitation of these studies, which possibly accounts for the varying results, is that they have been based on different clinical and non-representative subsamples. Previous research on the association between personality factors and suicidality has also been criticized for a lack of controlling for potentially important covariates such as the presence of psychiatric disorders or certain sociodemographic characteristics known to influence suicide risk [3]. Although large scale research primarily used aggregated data to conduct state-level analyses of the association between personality dimensions and suicide rates [19,20], such study designs are incapable to adjust for individual psychiatric comorbidity.

### Aims of the study

Considering all of the above it was the aim of this study to assess the relationship between the Big Five personality dimensions and suicidality in a representative sample of the German general population while controlling for anxiety and depression and other possible confounding sociodemographic factors. To our knowledge, this is the first study to assess this association in a representative population-based sample.

## Methods

### Sample

A cross-sectional questionnaire survey of a randomly generated population sample in Germany was conducted through face-to-face contact by a demographic consulting company (USUMA, Berlin, Germany). The fieldwork was conducted in April and May 2011. Random multistage sampling procedures were used. Germany was divided into 258 sampling areas representing Eastern and Western parts as well as different (rural and urban) regions of the country. Members of households in the selected areas were randomly chosen. The inclusion criteria were that participants were aged at least 18, able to read and to understand German. The sample was designed to be representative of the German population in terms of age, gender, and education. The selected individuals were personally approached by interviewers, who collected the sociodemographic information face-to-face; the respondents then themselves anonymously filled out the self-report questionnaires. The interviewers did not read or evaluate the results. Contact was attempted for 4,386 subjects, of whom 4,327 fitted the criteria. A total of 2,555 subjects agreed to participate.

### Ethics Statement

All the participants volunteered and received a data protection declaration in agreement with the Helsinki Declaration. The study was approved by the Ethics Committee of the Medical Faculty of the University of Leipzig. It was also approved according to the ethical guidelines of the “German Professional Institutions for Social Research” [Arbeitskreis Deutscher Markt- und Sozialforschungsinstitute, Arbeitsgemeinschaft Sozialwissenschaftlicher Institute, Berufsverband Deutscher Markt und Sozialforscher].

### Measures

Detailed sociodemographic characteristics were collected from each study participant. The following psychometrically validated instruments were used to assess personality, anxiety, depression, and suicidality in the population sample.

### Big Five Inventory-10 (BFI-10) [21]

The BFI-10 is a short self-rating instrument based on the Big Five Inventory-44 [22] reducing the original number of items from 44 to 10 with two items measuring each of the five personality dimensions (extraversion, agreeableness, conscientiousness, neuroticism, openness). The German version of the instrument was used [21]. It was shown to have acceptable psychometric properties with a clear five factor structure and good external validity [21].

### GAD-7 [23]

The German version of the GAD-7 (original version [24]:) was used to measure anxiety symptoms. It consists of 7 items with total scores ranging from 0 to 21. A score of ≥ 10 has been identified as indicating a possible diagnosis of generalized anxiety disorder in the general population [23]. The GAD-7 was also shown to be useful in identifying people with panic disorder, social anxiety disorder, and posttraumatic stress disorder [25]. It was shown to have high internal consistency and reliability [23]. For the purpose of this study a cut-off score of ≥ 10 was used to identify participants with moderate or severe anxiety symptoms.

### Patient Health Questionnaire – 9 (PHQ-9) [26]

Depression was assessed using the German version of the depression scale of the PHQ [27]. Each of its nine items is scored on a four-point Likert scale and describes one of the DSM-IV criteria for major depressive disorder. Numerous studies have shown excellent validity and reliability of the PHQ-9 [28,29]. A cut-off score of ≥ 10 has been recommended for detecting the presence of severe depressive symptoms and has been used in our study [28].

### Suicidal Behaviors Questionnaire-Revised (SBQ-R) [30]

Suicidality was assessed using the SBQ-R. It is a short, 4-item self-report questionnaire measuring different dimensions of suicidality: lifetime suicide ideation and / or suicide attempt, frequency of suicidal ideation over the past twelve months, threats of suicide attempt, and self-reported likelihood of suicidal behavior in the future. It has been validated in the adult general population and showed good internal consistency and reliability [30]. A cut-off score of ≥ 7 was recommended for use in general population samples for identifying groups at risk of suicide. The validity of the SBQ-R was established by differentiating between individuals with suicide-risk status from non-suicidal groups. Suicide-risk status was defined as past or present suicidal behavior (i.e. suicide attempts or suicide ideation). The SBQ-R showed very good sensitivity (93%) and specificity (95%), a positive predictive value of 70% and a negative predictive value of 99% for identifying individuals with suicide-risk status [30]. The SBQ-R was translated into German and back-translated by a native speaker. The German version of the SBQ-R in the present study had adequate internal consistency (α = 0.76) [31].

### Statistics

All analyses were calculated using IBM SPSS Statistics 21. Chi-square tests were calculated for group differences for categorical variables and independent *t*-tests for metric variables. Binary logistic regression analyses were conducted to assess predictors of suicide risk with multivariate models incorporating covariates. Alpha for all analyses was *p* < 0.05 and all reported *p*-values are two-tailed.

## Results

In total, 2555 persons participated in the study. Due to missing data in one of the key instruments (BFI-10, SBQ-R, PHQ-9, GAD-7) 128 participants had to be excluded in this study. The final dataset comprised 2427 participants with a mean age of 49.3 years (SD±18.1, range: 14-97). Further sample characteristics are shown in table 1.

**Table 1:** Sample characteristics.

		n (%)
Sex	Female	1274 (52.5%)
	Male	1153 (47.5%)
East vs. West of Germany	East of Germany	473 (19.5%)
	West of Germany	1954 (80.5%)
Urbanity	Rural area	2123 (87.5%)
	Urban area	304 (12.5%)
Family Status	Married, living together	1174 (48.4%)
	Married, living apart	38 (1.6%)
	Umarried	634 (26.1%)
	Divorced	297 (12.2%)
	Widowed	284 (11.7%)
Religious Denomination	Protestant	956 (39.4%)
	Catholic	796 (32.8%)
	Other	75 (3.1%)
	No denomination	545 (22.5%)
	No information	55 (2.3%)
Employment status	Employed	1208 (49.8%)
	Unemployed	169 (7.0%)
	Retired	731 (30.1%)
	Homemaker	105 (4.3%)
	In training / schooling	211 (8.7%)
	No information	3 (0.1%)
Household income	<1250€/month	567 (23.4%)
	1250 to 2500€/month	1094 (45.1%)
	>2500€/month	698 (28.8%)
	No information	68 (2.8%)

Mean scores of the PHQ-9, GAD-7, and SBQ-R and prevalence rates of depression, anxiety, and suicidality for the whole sample and separated by sex are given in table 2. Significant sex differences were observed for the mean scores of the PHQ-9, GAD-7, and SBQ-R and for the presence of anxiety. The scores of the BFI-10 are shown in table 3. Significant sex differences were also found in the personality dimensions agreeableness, conscientiousness, and neuroticism.

**Table 2:** Prevalence of depression, anxiety, and suicide risk.

		Whole sample	Male	Female	Statistics	
					T	*p*
Depression (PHQ-9)	Mean scores	3.22 (SD ±3.92)	2.83 (±3.63)	3.58 (SD ±4.14)	-4.69	<0.001
	Depression (PHQ ≥ 10)	189 (7.8%)	80 (6.9%)	109 (8.6%)		0.079
	No depression (PHQ < 10)	2238 (92.2%)	1073 (93.1%)	1165 (91.4%)		
Anxiety (GAD-7)	Mean scores	2.51 (SD ±3.05)	2.22 (SD ±2.78)	2.78 (SD ±3.26)	-4.52	<0.001
	Anxiety (GAD ≥ 10)	108 (4.4%)	4ß (3.5%)	68 (5.3%)		0.016
	No anxiety (GAD < 10)	2319 (95.6%)	1113 (96.5%)	1206 (94.7%)		
Suicidality (SBQ-R)	Mean scores	3.79 (SD ±1.77)	3.71 (SD ±1.56)	3.86 (SD ±1.94)	-2.11	0.035
	Suicide risk (SBQ-R ≥ 7)	112 (4.6%)	47 (4.1%)	65 (5.1%)		0.134
	No suicide risk (SBQ-R < 7)	2315 (95.4%)	1106 (95.9%)	1209 (94.9%)		

**Table 3:** Sex Differences in Personality factors.

		Whole sample	Male	Female	Statistics	
					T	*p*
Personality factors	Extraversion	3.40 (SD ±0.9)	3.44 (SD ±0.88)	3.36 (SD ±0.92)	2.20	0.028
	Agreeableness	3.38 (SD ±0.72)	3.29 (SD ±0.74)	3.46 (SD ±0.7)	-5.85	<0.001
	Conscientiousness	3.96 (SD ±0.81)	3.93 (SD ±0.83)	3.98 (SD ±0.79)	-1.39	0.165
	Neuroticism	2.57 (SD ±0.8)	2.43 (SD ±0.76)	2.70 (SD ±0.81)	-8.22	<0.001
	Openness	3.17 (SD ±0.88)	3.05 (SD ±0.88)	3.30 (SD ±0.86)	-7.08	<0.001

The logistic regression model with suicide risk (SBQ-R score ≥ 7) as the dependent variable is shown in table 4. The Hosmer-Lemeshow-test indicated a satisfactory model fit (χ^2^=4.41, *p*=0.82) and the correlation matrix showed no signs of multicollinearity. The personality factors extraversion, conscientiousness, neuroticism, and openness, and the presence of depression were significantly associated with suicidality.

**Table 4:** Logistic regression analysis of suicide risk (whole sample, n = 2427).

	*B*	S.E.	Wald	OR	95% CI	*p*
**Extraversion**	**-0.43**	**0.13**	**10.15**	**0.65**	**0.5-0.85**	**0.001**
Agreeableness	-0.74	0.15	0.25	0.93	0.7-1.24	0.62
**Conscientousness**	**-0.35**	**0.14**	**6.44**	**0.71**	**0.54-0.92**	**0.011**
**Neuroticism**	**0.49**	**0.15**	**10.41**	**1.64**	**1.21-2.2**	**0.001**
**Openness**	**0.33**	**0.14**	**5.88**	**1.39**	**1.07-1.82**	**0.015**
**Depression**	**1.4**	**0.31**	**20.85**	**4.04**	**2.22-7.37**	**<0.001**
Anxiety	0.58	0.36	2.67	1.79	0.89-3.6	0.1
Sex	-0.05	0.24	0.05	0.95	0.6-1.52	0.83
Age	-0.01	0.01	1.05	0.99	0.97-1.01	0.31
Family status			1.85			0.76
Religion			1.65			0.65
Urbanity	-0.09	0.33	0.08	0.91	0.48-1.74	0.78
East *vs.* West	-0.12	0.31	0.14	0.89	0.48-1.65	0.71
Employment status			7.81			0.1
Household income			2.84			0.24
Constant	-2.7	1.26	4.57	0.07		0.03

Next, the results of the logistic regression analyses performed separately for males and females are shown in tables 5 and 6. For males, extraversion, conscientiousness, and the presence of anxiety were significantly associated with suicidality. The model fit was satisfactory (Hosmer-Lemeshow-test: χ^2^=7.24, *p*=0.51) and no signs of multicollinearity were detected. In the model for females, neuroticism, openness, and the presence of depression were significantly associated with suicidality. Again, the model fit was satisfactory (Hosmer-Lemeshow-test: χ^2^=9.89, *p*=0.27) and no multicollinearity was detected.

**Table 5:** Logistic regression analysis of suicide risk for males (n=1153).

	*B*	S.E.	Wald	OR	95% CI	*p*
**Extraversion**	**-0.62**	**0.2**	**9.4**	**0.54**	**0.36-0.8**	**0.002**
Agreeableness	-0.11	0.23	0.23	0.9	0.58-1.4	0.63
**Conscientousness**	**-0.66**	**0.21**	**10.19**	**0.52**	**0.35-0.78**	**0.001**
Neuroticism	0.03	0.24	0.01	0.98	0.61-1.57	0.92
Openness	0.15	0.21	0.54	1.16	0.78-1.74	0.46
Depression	0.92	0.5	3.42	2.52	0.95-6.71	0.06
**Anxiety**	**1.29**	**0.58**	**4.99**	**3.63**	**1.17-11.26**	**0.03**
Age	-0.03	0.02	2.2	0.97	0.94-1.01	0.14
Family status			3.33			0.5
Religion			0.87			0.83
Urbanity	-0.33	0.51	0.41	0.72	0.27-1.97	0.52
East *vs* West	-0.23	0.44	0.26	1.25	0.53-2.99	0.61
Employment status			7.74			0.1
Household income			2.9			0.24
Constant	1.83	1.95	0.88	6.21		0.35

**Table 6:** Logistic regression analysis of suicide risk for females (n=1274).

	*B*	S.E.	Wald	OR	95% CI	*p*
Extraversion	-0.3	0.19	2.55	0.74	0.52-1.07	0.11
Agreeableness	-0.16	0.21	0.57	0.86	0.57-1.28	0.45
Conscientousness	-0.15	0.2	0.58	0.86	0.58-1.27	0.45
**Neuroticism**	**0.94**	**0.21**	**19.41**	**2.57**	**1.69-3.91**	**<0.001**
**Openness**	**0.55**	**0.19**	**8.13**	**1.73**	**1.19-2.53**	**0.004**
**Depression**	**2.1**	**0.41**	**25.61**	**8.14**	**3.61-18.33**	**<0.001**
Anxiety	-0.13	0.5	0.07	0.88	0.33-2.33	0.8
Age	-0.001	0.02	0.001	0.99	0.97-1.03	0.97
Family status			2.05			0.73
Religion			3.99			0.26
Urbanity	0.29	0.49	0.36	1.34	0.51-3.52	0.55
East *vs* West	-0.29	0.48	0.36	0.75	0.29-1.92	0.55
Employment status			1.77			0.78
Household income			1.87			0.39
Constant	-6.62	1.74	14.4	0.001		<0.001

## Discussion

This study of the relationship between personality dimensions based on the Big Five model and suicidality in a representative sample of the German general population yielded several noteworthy findings. An increased suicide risk indicated by a SBQ-R score ≥ 7 [30] was detected in 4.6% (n=112) of the respondents. The SBQ-R is an instrument which taps into different dimensions of suicidality including current and lifetime suicide ideation and attempt and self-reported likelihood of suicidal behavior in the future [30]. Therefore, a direct comparison with studies measuring only one aspect of suicidality is not recommendable. Nevertheless, similar results measuring suicidality in general population samples have recently been reported. A recent German population based study found a prevalence of suicidal ideation of 8.0% [32] and a European study reported a prevalence of 7.2% for lifetime suicide ideation and 1.3% for suicide attempts [33].

The multivariate logistic regression analyses showed that neuroticism and openness are associated with suicidality, while extraversion and conscientiousness are inversely associated with suicide-related behavior, thus posing protective factors. Overall, these findings corroborate previous research on the association between the big five personality dimensions and suicidality. Neuroticism, a personality trait frequently associated with negative affectivity and maladaptive coping strategies [34,35], has consistently been found to be associated with increased suicidality [3,12,13,14,15,18]. Likewise, extraversion has regularly been associated with reduced suicidal behavior [3,14,18]. Extraversion has been described as a tendency towards positive affect and low levels of extraversion are related to hopelessness and a negative outlook on life [34,36]. There is less evidence for the impact of the remaining personality dimensions on suicidality. In line with our results, openness was reported to be associated with suicide ideation in a sample of elderly depressed patients [12]. The authors argued that on the one hand openness may increase the likelihood of reporting suicide ideation as measured by self-report instruments and on the other hand it may decrease actual risk of death by suicide, presumably by increasing the chance of clinical intervention through timely reporting of suicidality [12]. High levels of openness have also been linked to cognitive distortion, lack of insight, and impulsivity, which could also explain the association with suicide-related behaviors in our sample [37,38]. Finally, low levels of conscientiousness have been found to be associated with increased suicidal ideation in previous studies [14,17]. There is evidence that low levels of conscientiousness are related to impulsive tendencies and substance use disorders as well as deficits in active coping strategies [8,39,40], which might explain increased suicide risk in these individuals.

The presence of moderate to severe depressive symptoms was the only other variable with a significant impact in the multivariate regression model. Depression is a well-known risk factor for suicidality [41]. No other variables showed a significant association with suicide risk including well established risk factors for completed suicide such as male sex, older age, or unemployment [1]. Additionally, it can be hypothesized that there are characteristic differences between suicide completers and persons with elevated suicide risk as assessed by self-report questionnaires such as the SBQ-R in our study. For example, it is known, that women are more likely to engage in nonfatal suicide attempts while the risk for completed suicide is greater in men [42,43,44].

In the separate analyses for males and females a distinctive pattern of personality factors was associated with suicide risk. For males, low levels of extraversion and low levels of conscientiousness were significant predictors. Velting (1999) also reported low levels of conscientiousness to be associated with suicidal ideation and a facet level analysis of the conscientiousness personality dimension identified lack of self-discipline as the most powerful predictor of suicidal ideation in men. On the other hand, low extraversion has not been previously reported to be specifically associated with male suicide risk, but is a well-established risk factor for both sexes.

Interestingly, high anxiety levels were associated with suicide-related behaviors in the male population, an association not found in women. These findings are in contrast to previous research on the association between anxiety disorders and suicidality, which found anxiety disorders to be an independent risk factor for suicide ideation and attempt for both sexes [45,46]. Cougle et al. (2009) report that for women all four anxiety disorders (social anxiety disorder, posttraumatic stress disorder, generalized anxiety disorder, panic disorder) were predictive of suicidality, while for men only posttraumatic stress disorder and panic disorder were identified as risk factors.

In our study, suicide-related behavior of females was predicted by high levels of neuroticism and openness and the presence of moderate to severe depressive symptoms. Velting (1999) also found neuroticism to be associated with suicide ideation in females. Neuroticism has strong links with a vulnerability for the development of depressive episodes and the prevalence of depression is known to be substantially higher in women [47,48]. This interaction could possibly explain the significance of neuroticism as a risk factor for suicide in women but not in men. High levels of openness together with high levels of neuroticism could be described as a personality style reflecting impulsive behavior and maladaptive, ineffective coping strategies [37,38,40]. These maladaptive strategies could help to explain greater suicide risk especially when combined with a tendency for negative affectivity (high levels of neuroticism).

In summary, the findings of this study highlight the role of personality dimensions as risk factors for suicide-related behaviors. Different personality dimensions are significantly associated with suicide-related behaviors even when adjusting for other known risk factors of suicidality. In the multidimensional diathesis-stress model of suicide [1], these personality features can be conceptualized as representing a trait-like vulnerability for suicide-related behaviors. While these personality factors are arguably of only marginal clinical utility in the assessment of acute suicidality, they may constitute an important component of a comprehensive evaluation of individuals at long-term risk of suicide-related behaviors [49]. Since these personality dimensions are known to be rather stable over time, they underscore the necessity of more intensive psychological or psychotherapeutic treatments of at-risk individuals, e.g. by focusing on maladaptive coping strategies, which are characteristic for certain combinations of personality factors. These findings may also serve as a base for future research into neurobiological mechanisms underlying these relationships [50].

### Limitations

While a response rate of 58.3% is relatively high for a population-based survey, a possible bias of the respondents cannot be ruled out, which has to be taken into account when generalizing our findings. Due to time constraints in this population-based study, the short BFI-10 was used to assess personality, which does not allow a more in depths analysis of the facets that constitute the higher- order personality dimensions. Further research taking into account sex differences in the interaction between personality variables and suicide risk is warranted. Another limitation of this study is its reliance on self-reported suicidality, even though there is evidence that shows high-levels of agreement between self-report suicide assessment and face-to-face interviews [51]. The respondents anonymously filled out the self-report suicide questionnaires and the interviewers did not read or evaluate the results. There is a risk that suicidal participants were not immediately identified and provided with professional support. Further research should consider this ethical issue in order to distinguish between the research participants’ right to privacy and uncovering suicide ideation among respondents [52]. Researchers should carefully assess the value of anonymous answers versus the welfare of the participants. The SBQ-R is a well-validated and commonly used tool to assess suicidality. But the ability of the SBQ-R to predict future suicide attempts or completed suicide has not been established in prospective longitudinal studies yet. Finally, due to the cross-sectional design of this study, no causal conclusions can be inferred from the results. Longitudinal research into the association of personality characteristics and suicide risk with the inclusion of completed suicides is needed.
